# Adaptation of mitochondrial bioenergetics to coenzyme Q deficiency in human endothelial cells after chronic exposure to bisphosphonates

**DOI:** 10.1038/s41598-025-02710-8

**Published:** 2025-05-22

**Authors:** Adrianna Budzinska, Lukasz Galganski, Krzysztof Wojcicki, Wieslawa Jarmuszkiewicz

**Affiliations:** Mitochondrial Biochemistry Research Group, Laboratory of Mitochondrial Biochemistry, Faculty of Biology, Collegium Biologicum, Adam Mickiewicz University, Uniwersytetu Poznanskiego 6, 61‑614 Poznan, Poland

**Keywords:** Alendronate, Bisphosphonates, Coenzyme Q, Endothelial cells, Mitochondrial respiration, Zoledronate, Biochemistry, Cell biology

## Abstract

Nitrogen-containing bisphosphonates (N-BPs), widely used in bone disease therapy, inhibit the mevalonate pathway, which affects coenzyme Q (CoQ) biosynthesis and may compromise mitochondrial function, particularly in endothelial cells where oxidative stress and mitochondrial dysfunction contribute to cardiovascular disease. This study examined the effects of chronic six-day exposure of human endothelial cells to N-BPs on mitochondrial bioenergetic functions, focusing on drug-induced mitochondrial CoQ (mtCoQ) deficiency. Compared with the mitochondria of control cells, those of endothelial cells treated with 5 µM alendronate or 1 µM zoledronate presented a significant 45–50% decrease in total mtCoQ pool, loss of reduced (mtCoQH_2_) antioxidant mtCoQ pool, and elevated mitochondrial antioxidant protein superoxide dismutase 2 (SOD2) and uncoupling protein 2 (UCP2) levels. Exposing endothelial cells to N-BPs also led to an overall reduction in mitochondrial substrate oxidation, except for increased fatty acid oxidation. Additionally, the mitochondria of N-BP-treated endothelial cells presented decreased respiratory rates, membrane potential, and ATP synthesis efficiency, and increased H_2_O_2_ production resulting from increased mtCoQ reduction during the oxidation of complex I (CI) and CII substrates. N-BP-induced mtCoQ deficiency also resulted in rearranged respiratory chain supercomplexes, particularly downregulation of the III_2_ + IV supercomplex, and decreased CII, CIII, and CV protein levels and activities. Despite the N-BP-induced decrease in *a*-heme levels, maximal CIV activity remained unaffected in endothelial mitochondria. These findings highlight the role of N-BPs in disrupting mtCoQ redox homeostasis and associated bioenergetic functions in endothelial mitochondria.

**Supplementary Information:**

The online version contains supplementary material available at 10.1038/s41598-025-02710-8.

## Introduction

Mitochondria play a key role in cellular bioenergetics, containing the respiratory chain responsible for ATP production through oxidative phosphorylation (OXPHOS), the final step in aerobic respiration. Coenzyme Q (CoQ) is a critical electron carrier in the mitochondrial respiratory chain and an important antioxidant found in all cell membranes^1^. Nevertheless, mitochondrial CoQ (mtCoQ) also generates mitochondrial reactive oxygen species (mtROS) within the respiratory chain. mtCoQ is directly involved in the formation of superoxide/hydrogen peroxide at four mtCoQ-binding sites, i.e., the mtCoQ-reducing site of complex I (I_Q_) (both during forward and reverse electron transfer)^2,3^, mitochondrial glycerol-3-phosphate dehydrogenase (G_Q_)^4^, dihydroorotate dehydrogenase (D_Q_)^5^, and the mtQH_2_-oxidizing site of complex III (III_Qo_)^6^. Mitochondrial dysfunction, including that associated with decreased CoQ levels, has been linked to cellular dysfunction and several metabolic, cardiovascular, and neurodegenerative diseases^7,8^.

Bisphosphonates, synthetic analogs of pyrophosphate, are frequently used to manage osteoporosis and other bone disorders because they suppress bone resorption mediated by osteoclasts^9,10^. Bisphosphonates are divided into non-nitrogen-containing and nitrogen-containing bisphosphonates (N-BPs)^11^. N-BPs can be further divided into second-generation (including alendronate) and more efficient third-generation (including zoledronate) drugs. N-BPs target and inhibit farnesyl diphosphate synthase (FPPS), a critical enzyme in the intracellular mevalonate pathway, thereby interfering with the prenylation/activation of small GTPases necessary for osteoclast survival and functionality^9–11^. The mevalonate pathway is a crucial metabolic route that provides isoprenoid units and plays a central role in the biosynthesis of important molecules, including cholesterol, steroid hormones, heme *a*, and CoQ^12,13^. Decreased mtCoQ levels due to inhibition of the mevalonate pathway may lead to dysfunctional mitochondrial respiration and increased oxidative stress, as has been described for statins, which are cholesterol-lowering drugs^1,14–17^. Statins inhibit the first step of the mevalonate pathway catalyzed by 3-hydroxy-3-methylglutaryl-coenzyme A (HMG-CoA) reductase and reduce the biosynthesis of isoprenoid intermediates, including those required for CoQ synthesis, leading to mtCoQ deficiency and impaired electron transport chain function, which has been described mainly in myocytes and endothelial cells^1,14–17^. N-BPs also inhibit the mevalonate pathway that supplies the isoprenoid unit necessary for CoQ synthesis; however, limited research has been conducted on the effect of N-BPs on cellular CoQ levels. N-BP therapy is associated with decreased plasma CoQ levels in postmenopausal women^18^. Furthermore, we recently demonstrated that the N-BPs alendronate and zoledronate significantly reduce total CoQ levels (i.e., CoQ from all membranes) in cultured human endothelial cells^19^.

Endothelial cells lining the blood vessel interior come into contact with circulating drugs, such as N-BPs. Alterations in blood serum composition can influence the oxidative metabolism of endothelial cells, potentially resulting in oxidative stress. Endothelial dysfunction is closely related to the overproduction of reactive oxygen species (ROS), especially mitochondrial ROS (mtROS), which may promote the development of cardiovascular diseases^20–23^. While N-BPs act primarily on the skeletal system, research indicates that these FPPS inhibitors may exert unintended effects on nonskeletal tissues and affect the endothelial cell functions crucial for maintaining vascular homeostasis and metabolism^24,25^. Long-term use of N-BPs or high doses may lead to adverse effects, including an increased risk of cardiovascular events or bisphosphonate-related osteonecrosis of the jaw due to the inhibition of angiogenesis^26,27^.

N-BPs, especially zoledronate, have been reported to affect endothelial cell migration, survival and apoptosis^28^. Moreover, in endothelial cells, N-BPs impede angiogenesis by disrupting protein prenylation, affecting endothelial cell adhesion, proliferation, survival, migration, and actin stress fibers^29–32^. We recently reported that alendronate and zoledronate influence cellular energy and redox status in endothelial cells by significantly decreasing cellular CoQ levels, alternating cellular respiration and mitochondrial turnover, reducing cellular ATP levels, and increasing ROS production^19^. Although we previously reported that N-BPs cause a deficiency in total CoQ in endothelial cells^19^, the present study is, to our knowledge, the first to investigate mtCoQ deficiency and its downstream bioenergetic consequences, including the redox state of mtCoQ, mtROS formation, and oxidative phosphorylation (OXPHOS) system remodeling. Understanding how N-BPs influence endothelial mitochondrial bioenergetic function is important for elucidating their potential effects on the cardiovascular system.

This study investigated the effects of chronic exposure to alendronate and zoledronate on mitochondrial bioenergetic functions in cultured human endothelial cells, focusing on N-BP-induced mtCoQ deficiency. We measured mitochondrial respiratory activities, membrane potential (ΔΨ), OXPHOS efficiency, uncoupling, mtROS production, mtCoQ content and reduction levels, and the molecular organization of OXPHOS components to elucidate whether prolonged treatment with N-BPs induces mitochondrial adaptations and how they may be linked to mtCoQ availability.

## Materials and methods

### Cell culture

Experiments were conducted using the EA.hy926 human endothelial cell line (ATCC CRL-2922, RRID: CVCL_3901, ATCC, Manassas, VA, USA) derived from the human umbilical vein. The cells were cultured for six days under standard conditions or exposed to N-BPs, specifically 5 µM alendronate or 1 µM zoledronate, following previously established protocols^19^.

### Mitochondria isolation

Mitochondria were isolated as previously described^15,33^ with minor modifications. After six days of culture, the cells from the control and N-BP-treated groups were harvested using trypsin/ethylenediaminetetraacetic acid (EDTA). The cells were then washed with 10% FBS (in phosphate-buffered saline, [PBS]), 5% FBS, and PBS alone. The samples were subsequently centrifuged at 1,200 × *g* for 10 min at 4 °C. The resulting cell pellets were resuspended in medium (PREPI) comprising 0.25 M sucrose, 1.5 mM EDTA, 1.5 mM ethylene glycol bis(2-aminoethyl)tetraacetic acid, 0.2% BSA, and 5 mM Tris/HCl (pH 7.2). The cells were homogenized via 17 passes with a Dounce homogenizer. After the homogenates were centrifuged at 1,200 × *g* for 10 min, the pellet was resuspended, subjected to additional homogenization (12 passes), and centrifuged again to extract the remaining mitochondria. The supernatants with crude mitochondria were then centrifuged at 12,000 × *g* for 10 min. The mitochondrial pellets were washed with PREPII medium (0.25 M sucrose and 15 mM Tris/HCl [pH 7.2]) and centrifuged again at 12,000 × *g* for 10 min. The final mitochondrial pellets were resuspended in PREPII medium.

### ∆Ψ and mitochondrial respiration

∆Ψ and mitochondrial respiration in isolated endothelial mitochondria were assessed as previously described^15,33^. ΔΨ was recorded simultaneously with oxygen consumption via a tetraphenylphosphonium (TPP)-specific electrode. Oxygen uptake was determined polarographically via a Rank Bros. (Cambridge, UK) oxygen electrode or a Hansatech (King’s Lynn, UK) oxygen electrode. Measurements were conducted in 0.6 or 3.0 mL of a standard incubation medium (20 mM Tris/HCl, pH 7.2, 150 mM sucrose, 4 mM MgCl_2_, 2.5 mM KH_2_PO_4_, and 0.1% BSA) with either 0.5 or 2.5 mg of mitochondrial protein, respectively, at 37 °C.

Phosphorylating (state 3) respiration was assessed with either 150 µM ADP (ADP/O measurements) or 1.7 mM ADP (maximal phosphorylating respiration), whereas uncoupled respiration was assessed with up to 0.9 µM carbonyl cyanide-p-trifluoromethoxyphenylhydrazone (FCCP). Non-phosphorylating (state 4, resting state) respiration was measured without ADP. The respiratory substrates used were 4 mM malate (complex I, CI, substrate), 4 mM succinate plus 1.2 µM rotenone (CII substrate), 4 mM succinate plus 4 mM malate, 0.4 mM duroquinol plus 1.2 µM rotenone (CIII activity measurements), 4 mM glutamate, and 20 µM palmitoylcarnitine plus 2 mM carnitine.

The maximal activity of complex IV (CIV, cytochrome *c* oxidase [COX]) was assessed by measuring the oxygen consumption rate using 0.2 mg of mitochondrial protein (0.33 mg/mL). The assay involved the sequential addition of 5 µM antimycin A, 5 mM ascorbate, 0.04% cytochrome *c*, and up to 1 µM *N*,*N*,*N*’,*N*’-tetramethyl-*p*-phenylenediamine.

### UCP and mitoBK_Ca_ activity

The activity of the uncoupling protein (UCP) or high-conductance Ca^2+^-activated mitochondrial potassium channel (mitoBK_ca._) in response to the driving force was quantified by measuring the oxygen consumption rate’s dependence on ∆Ψ during respiratory chain inhibition titration^33^. The respiratory rate was progressively decreased by inhibiting malate and succinate oxidation with increasing concentrations of rotenone (a complex I inhibitor) and malonate (a complex II inhibitor). The inhibitors were introduced in two stages: first, 0.3 µM rotenone combined with 1 mM malonate, followed by 0.6 µM rotenone paired with 2 mM malonate. Non-phosphorylating respiration was measured in the presence of ATP synthase and ATP/ADP translocase inhibitors (1 µg/mL oligomycin and 1 µM carboxyatractyloside, respectively) to eliminate ATP turnover-dependent proton leakage. For UCP activity measurements, carboxyatractyloside also prevented inducible fatty acid-mediated proton leakage through ATP/ADP translocase. UCP activity was determined by using 8 µM linoleic acid as an activator and 2 mM GTP as an inhibitor. MitoBK_ca_ activity was assessed by using 1 µM NS11021 as an activator and 2 µM iberiotoxin (IbTx) as an inhibitor. The linoleic acid-induced GTP-inhibited UCP activity and NS11021-induced IbTx-inhibited mitoBK_Ca_ activity were determined at the highest common ΔΨ value from the flux-force kinetic relationship.

### Mitochondrial CoQ content and reduction level

The mtCoQ content and reduction level were measured via extraction method followed by high-performance liquid chromatography analysis with a LiChrosorb RP-18 (10 μm) column and a GE Acta Explorer system (GE Healthcare, Chicago, IL, USA)^34^. Both oxidized (290 nm) and reduced (275 nm) forms of coenzyme Q10 (CoQ10) were detected. Commercial CoQ10 was used for peak calibration. The reduction level of mtCoQ (mtCoQH_2_/mtCoQtot), which indicates the proportion of reduced mtCoQ to total mtCoQ in isolated endothelial mitochondria during steady-state respiration, was calculated. Samples for mtCoQ extraction were collected while monitoring oxygen consumption and changes in ∆Ψ.

### Mitochondrial H_2_O_2_ production

Mitochondrial H_2_O_2_ production was measured using the Amplex Red assay (Invitrogen, Waltham, MA, USA) in a 24-well plate, read with a Tecan multimode reader (Tecan Group Ltd., Männedorf, Switzerland). The assay was conducted at 37 °C in 0.5 mL of a standard incubation medium containing 0.1 U/mL horseradish peroxidase, 5 µM Amplex Red, and 1 U/mL superoxide dismutase (SOD) with 0.5 mg mitochondrial protein (1 mg/mL). Fluorescence was recorded over 35 min at 545 nm (excitation) and 590 nm (emission). The mitochondria were exposed to respiratory substrates (4 mM malate, 4 mM succinate plus 1.2 µM rotenone, and 4 mM malate plus 4 mM succinate) with or without 1.5 mM ADP (phosphorylating and non-phosphorylating conditions, respectively). Known H_2_O_2_ concentrations were used for calibration to determine the rate of H_2_O_2_ production.

### Cytochrome *a* + *a*_3_ reduction

Endothelial mitochondria were resuspended at a final concentration of 5 mg protein/mL in a buffer (120 mM KCl, 10 mM Tris/HCl [pH 7.2], and 2.6 mM cyanide) and transferred to either the experimental or reference cuvette. Dithionite (0.15%) was added to the experimental cuvette (complete reduction), and K_3_[Fe(CN)_6_] (1 mM) was added to the reference cuvette (complete oxidation). The reduced minus oxidized difference spectra of cytochromes *a* + *a*_3_ were determined using a Shimadzu 1620 UV spectrophotometer (580–605 nm).

### Mitochondrial protein level immunodetection

Proteins from isolated mitochondria were separated using 8–11% sodium dodecyl sulfate-polyacrylamide gel electrophoresis (SDS–PAGE). Abcam (Cambridge, UK) primary antibodies were used to immunodetect the following proteins: cytochrome *c* oxidase subunit II (COXII; 20 kDa; ab110258), citrate synthase (CS; 46 kDa) (ab96600)uncoupling protein 2 (UCP2; (33 kDa; ab97931), uncoupling protein 3 (UCP3; 34 kDa; ab3477), coenzyme Q-binding protein CoQ10 homolog B (CoQ10B; 27 kDa; ab41997), acyl-coenzyme A dehydrogenase (ACADS; 41 kDa; ab156571), glutamate dehydrogenase (GDH; 61 kDa; ab89967), and total OXPHOS human WB antibody cocktail (ab110411), which contains antibodies against subunits of CI (18 kDa subunit NADH: ubiquinone oxidoreductase subunit B8 [NDUFB8]), CII (36 kDa subunit succinate dehydrogenase complex iron sulfur subunit B [SDHB]), CIII (subunit Core 2, 42 kDa), CIV (COXII; 20 kDa), and ATP synthase (complex V [CV] subunit α, 52 kDa). The anti-superoxide dismutase 2 (SOD2; 25 kDa) antibody (ADI-SOD) was purchased from Enzo Life Sciences (Farmingdale, NY, USA). Alomone Labs (Jerusalem, Israel) antibodies raised against the mitoBK_Ca_ subunits KCa1.1 (105 kDa; APC-107) and sloβ2 (42 kDa; APC-034) were used. The protein levels of COXII or CS were used as loading controls. Images of immunodetection results shown in the figures have been cropped to standardize the presentation, since the major loading control protein (COXII, 20 kDa) has a molecular mass similar to that of most of the proteins tested. The absence of images of adequate length in the figures is also due to the fact that after protein transfer the membranes were cut to allow separate immunodetection of target proteins and loading controls, due to the limited amount of mitochondrial material available. The original immunoblots can be found in Supplementary Information (Figs. S1–S5). Protein levels were digitally quantified using ImageJ software (US National Institutes of Health, Bethesda, MD, USA).

### BN–PAGE and in-gel activity assays

In-gel activity assays of complexes CI, CII, CIV, and CV following blue native polyacrylamide gel electrophoresis (BN–PAGE) separation (3–9.5% gradient gels) of mitochondrial proteins (100 µg) were conducted as previously described^15^. In addition, the BN-PAGE-separated proteins were transferred onto nitrocellulose membranes to determine the OXPHOS complexes by immunoblotting with an anti-UQCRC2 antibody (against CIII; ab14745, Abcam) or the Total OXPHOS Human WB Antibody Cocktail.

### Statistical analysis

The data are expressed as the means ± standard deviations (SD), and are based on a minimum of 4–8 independent mitochondrial isolations. Each measurement was conducted at least in triplicate. Significance was evaluated via ANOVA (followed by Tukey’s post hoc comparisons for *P* < 0.05) or nonparametric Kruscal–Wallis ANOVA (KW ANOVA) (followed by Dunn’s post hoc comparisons for *P* < 0.05) to determine statistically significant differences (*P < 0.05, **P < 0.01, ***P < 0.001).

## Results

For this study, we chose two representative N-BPs: zoledronate, recognized for its high potency attributed to its unique R2 side chain featuring a nitrogen-containing heterocyclic ring, and alendronate, characterized as a moderately potent N-BP^35^. The micromolar concentrations used in the experiments were based on published studies reporting the serum concentrations of these compounds in patients undergoing osteoporosis or bone cancer treatment after drug infusions^36–38^. For the experiments, human endothelial cells were cultured for six days with 5 µM alendronate or 1 µM zoledronate. We have previously shown that in endothelial cells at these concentrations, alendronate and zoledronate have no effect on cell viability but lead to a similarly significant reduction (by ~ 30%) in the cellular CoQ content^19^. Higher concentrations (from 7.5 µM for alendronate to 2.5 µM for zoledronate) significantly reduce endothelial cell viability and strongly (by ~ 60%) reduce the cellular CoQ content^19^.

### Total and reduced mtCoQ levels were significantly decreased, and SOD2 levels were increased in the endothelial mitochondria of N-BP-exposed cells

**Fig. 1:**
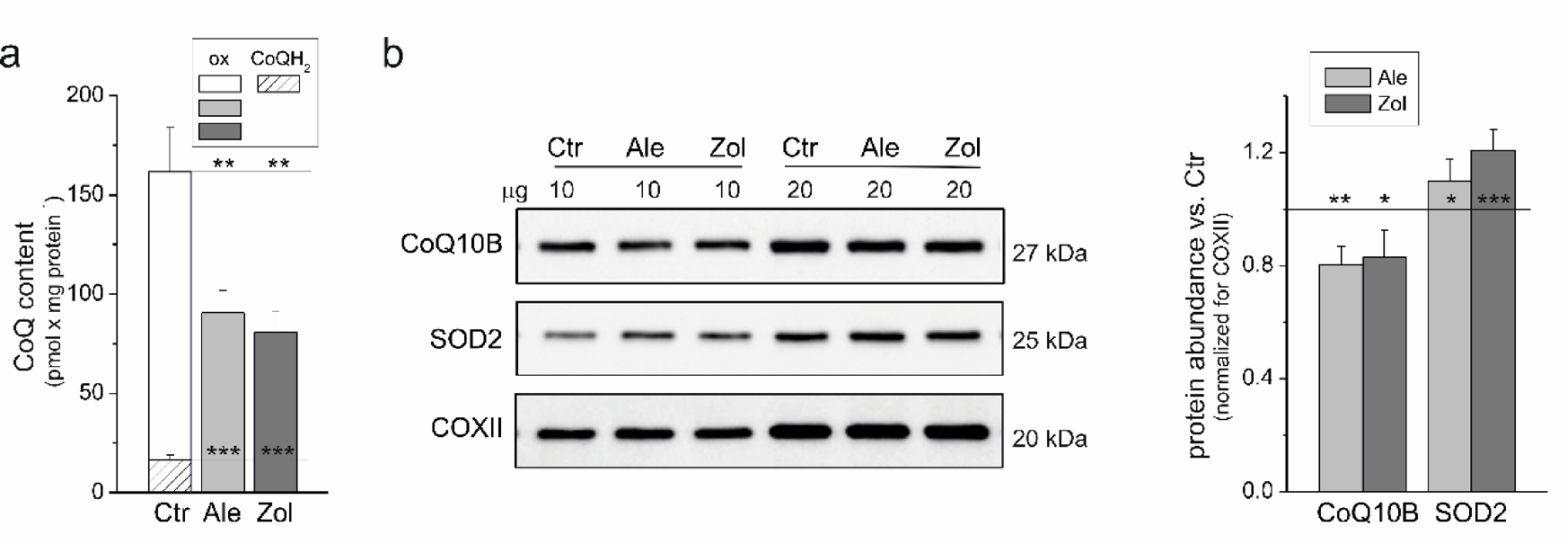
(**a**) mtCoQ content and (**b**) protein abundance of CoQ10B and SOD2 in mitochondria isolated from endothelial cells exposed for six days to 5 µM alendronate (Ale) or 1 µM zoledronate (Zol) compared with the mitochondria of control cells (Ctr). (**a**) The total (mtCoQH_2_ + mtCoQox), reduced (mtCoQH_2_), and oxidized (mtCoQox) CoQ pools were determined under fully oxidizing conditions (without respiratory mtCoQ-reducing substrates). Mean ± SD; *n* = 8; statistics: one-way ANOVA, *P* < 0.001 (***); *P* < 0.01 (**) relative to control mitochondria (horizontal lines). (**b**) Analyses of protein abundance accompanied by representative immunoblots. Loading control: COXII. The original immunoblots are shown in Fig. S1 (Supplementary Information). Mean ± SD; *n* = 5; statistics: KW ANOVA, *P* < 0.001 (***); *P* < 0.01 (**); *P* < 0.05 (*) relative to control mitochondria (horizontal lines).

To date, the impact of N-BPs on mtCoQ levels has not been studied extensively. We observed a 45–50% decrease in total mtCoQ content (mtCoQH_2_ + mtCoQ oxidized) in mitochondria isolated from endothelial cells cultured with 5 µM alendronate or 1 µM zoledronate compared with the mitochondria of control cells (Fig. 1a). Under fully oxidizing conditions (without respiratory mtCoQ-reducing substrates), we also measured the pool of reduced mtCoQ (mtCoQH_2_), which is not oxidizable by the respiratory chain and may act as an antioxidant pool^1^. The mtCoQH_2_ pool constituted ~ 12% of the total mtCoQ pool in the mitochondria of control cells and was not observed in the mitochondria of N-BP-treated cells. Furthermore, a significant ~ 20% decrease in the level of CoQ10B, which is required for mtCoQ function in the respiratory chain, was observed in the mitochondria of N-BP-treated cells (Fig. 1b). The disappearance of the reduced pool of mtCoQ (mtCoQH_2_) was accompanied by a 10–20% increase in the antioxidant enzyme SOD2 level.

mtCoQH_2_ is not only an important antioxidant but also a key electron carrier in the mitochondrial respiratory chain. Therefore, we next examined whether the decrease in mtCoQ observed after endothelial cell exposure to N-BPs influences mitochondrial respiration.

### Chronic six-day exposure of endothelial cells to N-BPs leads to an overall reduction in the oxidation of respiratory substrates in their mitochondria, except for increased fatty acid oxidation

**Fig. 2:**
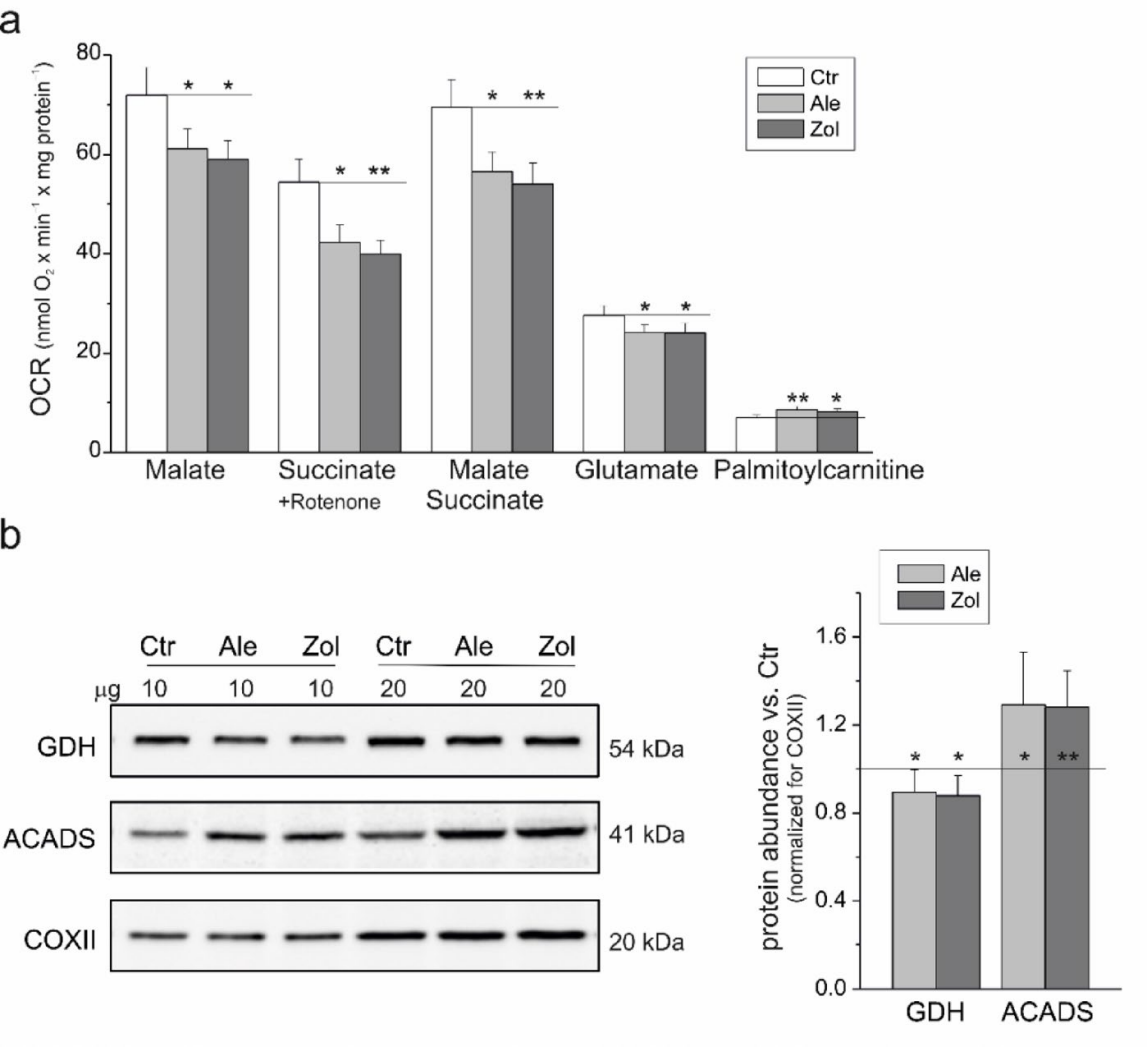
(**a**) Maximal respiratory chain activity (uncoupled respiration) with different substrates and (**b**) ACADS and GDH protein levels in mitochondria isolated from endothelial cells cultured with 5 µM alendronate (Ale) or 1 µM zoledronate (Zol) compared with the mitochondria of control cells (Ctr). (**a**) Uncoupled respiration in the presence of 0.8 µM FCCP; OCR, oxygen consumption rate. Mean ± SD; *n* = 8; statistics: one-way ANOVA, *P* < 0.01 (**); *P* < 0.05 (*) relative to control mitochondria (horizontal lines). (**b**) Analyses of protein abundance accompanied by representative immunoblots. Loading control: COXII. The original immunoblots can be found in Fig. S2 (Supplementary Information). Mean ± SD; *n* = 6; *P* < 0.01 (**); *P* 0.05 (*) relative to control mitochondria (horizontal lines).

We assessed the effects of treating endothelial cells with 5 µM alendronate or 1 µM zoledronate on mitochondrial respiratory function by measuring the maximal respiratory rate of isolated endothelial mitochondria via various respiratory substrates (Fig. 2a). Under uncoupling conditions, maximal oxidation of the weakest reducing substrate tested, palmitoylcarnitine, was increased by ~ 20% in the mitochondria of N-BP-treated endothelial cells compared with those of control cells. The enhanced oxidation of this substrate was accompanied by a similar increase in ACADS protein, the enzyme that catalyzes the initial step of fatty acid β-oxidation (Fig. 2b). This observation may indicate an increased share of fatty acids as energy fuels in oxidative metabolism in endothelial cells treated with N-BPs.

However, in the mitochondria of endothelial cells treated with alendronate or zoledronate, maximal glutamate oxidation and protein abundance of GDH, the enzyme responsible for the conversion of glutamate to α-ketoglutarate, which enters the tricarboxylic acid (TCA) cycle for oxidation, decreased slightly by ~ 13% relative to that in the mitochondria of control untreated cells (Fig. 2). Moreover, a significant reduction in the maximal oxidation of strongly reducing substrates (i.e., the CI substrate malate [~ 17%], the CII substrate succinate [~ 25%], and their mixture [~ 21%]) was observed (Fig. 2a). Therefore, we next examined the impact of these changes on mitochondrial ATP synthesis efficiency and ΔΨ.

### Respiratory rates, ΔΨ, and ATP synthesis efficiency during the oxidation of CI and CII substrates are reduced in the mitochondria of N-BP-treated endothelial cells

The functional bioenergetics parameters of endothelial mitochondria were compared during the oxidation of succinate alone (with rotenone), malate alone, and their mixture under non-phosphorylating and phosphorylating conditions. For all the tested substrates (Table 1), the chronic exposure of endothelial cells to alendronate or zoledronate decreased mitochondrial coupling parameters, the ADP/O ratio (by 13–16%), and the respiratory control ratios (by 13–19%), thus decreasing mitochondrial OXPHOS efficiency. Consequently, the ADP phosphorylation rate was significantly lower, albeit less significantly for malate alone (∼30%) and most significantly for succinate alone (∼40%), in the mitochondria of N-BP-treated cells compared to that in the mitochondria of control cells. These results indicate significantly reduced ATP synthesis in the mitochondria of N-BP-treated endothelial cells.

**Table 1:** Mitochondrial coupling parameters—the respiratory control ratio (RCR), the ADP/O ratio, and the rate of ADP phosphorylation (phosphorylating respiration × ADP/O)—in the mitochondria of control (Ctr) and alendronate (Ale)- or zoledronate (Zol)-treated endothelial cells.

	Malate			Succinate + Rotenone			Succinate + Malate		
	Ctr	Ale	Zol	Ctr	Ale	Zol	Ctr	Ale	Zol
RCR	4.28 ± 0.35	3.64 ± 0.31*	3.63 ± 0.34*	2.74 ± 0.23	2.30 ± 0.20*	2.31 ± 0.19*	3.46 ± 0.26	2.99 ± 0.23*	2.88 ± 0.23*
ADP/O	2.36 ± 0.18	1.99 ± 0.12*	2.05 ± 0.15*	1.32 ± 0.09	1.07 ± 0.08*	1.07 ± 0.08*	2.01 ± 0.17	1.67 ± 0.11*	1.69 ± 0.12*
Phosphorylation rate	163 ± 13	110 ± 8***	110 ± 10***	68.6 ± 5.6	39.6 ± 3.3***	38.3 ± 3.0***	135 ± 12	82.1 ± 6.1***	82.4 ± 6.6***

The phosphorylation rate is expressed in nmol ADP × min^−1^ × mg protein^−1^. Mean ± SD (*n* = 8); statistics: one-way ANOVA, *P < 0.05, *** *P* < 0.001 compared with control mitochondria.

**Fig. 3:**
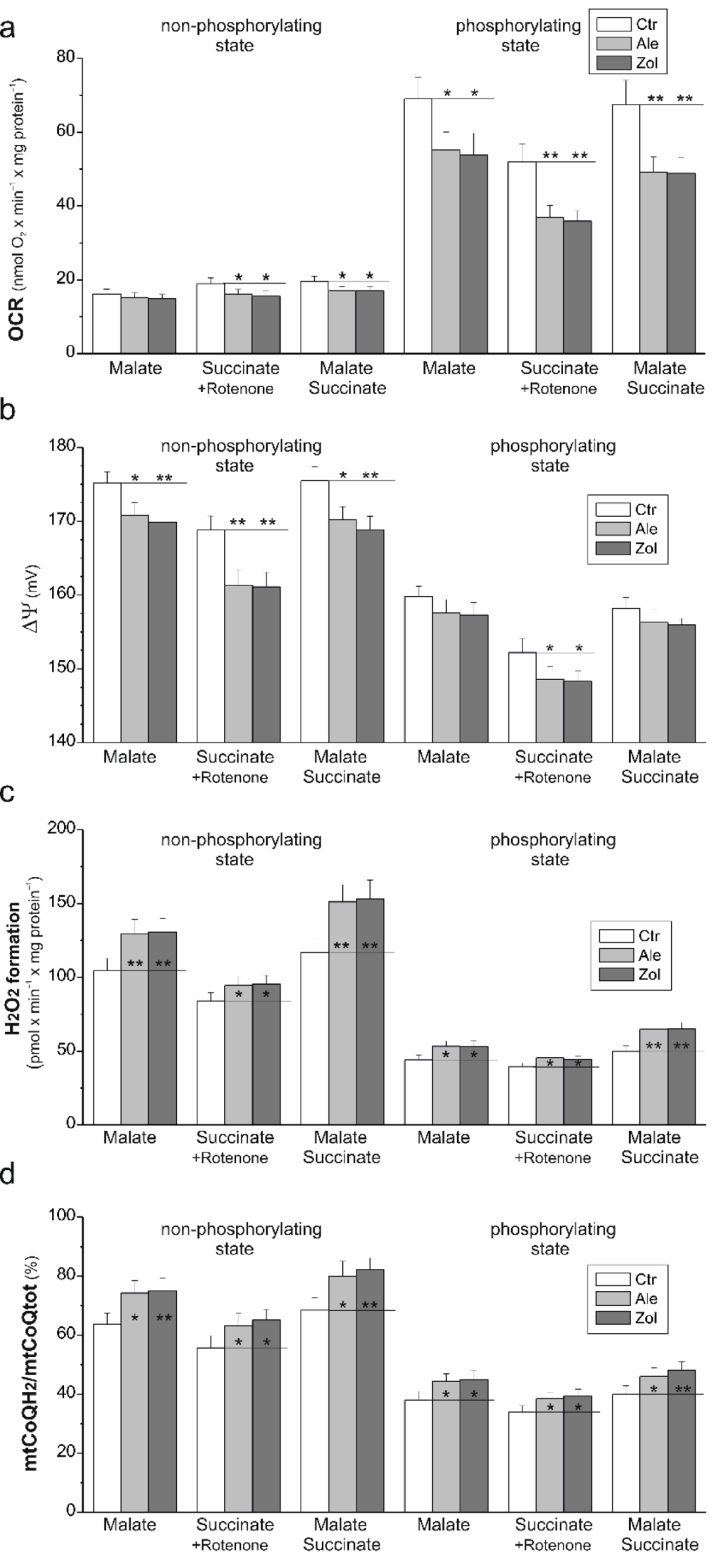
Functional parameters of mitochondria isolated from control (Ctr) and alendronate (Ale)- or zoledronate (Zol)-treated endothelial cells during the oxidation of malate, succinate (with rotenone), and a mixture of malate and succinate under non-phosphorylating and phosphorylating conditions. (**a**) Oxygen consumption rate (OCR). (**b**) ΔΨ. (**c**) H_2_O_2_ production rate. (**d**) mtCoQ reduction level (mtCoQH_2_/mtCoQtot). Mean ± SD; *n* = 6–8; statistics: one-way ANOVA, *P* < 0.01 (**); *P* < 0.05 (*) relative to control mitochondria (horizontal lines).

In contrast to non-phosphorylating conditions, under phosphorylating conditions, the respiratory rates with malate in the mitochondria of N-BP-treated cells were ~ 20% lower than those in the control cell mitochondria (Fig. 3a). Moreover, during succinate oxidation, reductions of ~ 17% and ~ 30% were observed under non-phosphorylating and phosphorylating conditions, respectively. Compared with that in the mitochondria of control cells, oxidation of the mixture of both substrates was also reduced in the mitochondria of N-BP-treated cells, and was less significant under non-phosphorylating (~ 13%) than phosphorylating (~ 26%) conditions. Additionally, under non-phosphorylating conditions, decreases in the respiration of CI and CII substrates were accompanied by decreases in ΔΨ (the largest during succinate oxidation; Fig. 3b). Under phosphorylating conditions, a statistically significant reduction in ΔΨ was observed only during the oxidation of succinate alone in the mitochondria of N-BP-treated cells compared with the mitochondria of control cells.

Overall, the mitochondria of endothelial cells cultured in the presence of alendronate or zoledronate presented a lower respiratory rate, ΔΨ, and OXPHOS efficiency than did the mitochondria of control cells. Notably, the observed changes were more pronounced during the oxidation of the CII substrate (succinate) than during the oxidation of the CI substrate (malate) when each substrate was oxidized individually.

mtCoQH_2_ is not only an important antioxidant but also plays a role in generating mtROS via the respiratory chain. Therefore, we investigated whether the decrease in mtCoQ observed after the exposure of endothelial cells to N-BPs affects the reduction level of mtCoQ and, thus, the production of mtROS.

### Chronic exposure of endothelial cells to N-BPs increases H_2_O_2_ production in mitochondria due to increased mtCoQ reduction level

In the mitochondria of N-BP-exposed endothelial cells relative to the mitochondria of control cells, H_2_O_2_ production was consistently greater under both respiratory conditions, regardless of the substrate (malate alone, succinate alone, or a combination of malate and succinate) (Fig. 3c). A comparison of H_2_O_2_ production during the oxidation of malate alone and of succinate alone revealed that these increases were more pronounced for the CI substrate (malate; ~22–25%) than for the CII substrate (succinate; ~13–15%). Moreover, in the mitochondria of N-BP-treated cells, the level of mtCoQ reduction consistently increased under both non-phosphorylating and phosphorylating conditions, regardless of the substrate (Fig. 3d). Likewise, these increases were more prominent during the CI substrate (~18%) versus CII substrate (~13%) oxidation.

**Fig. 4:**
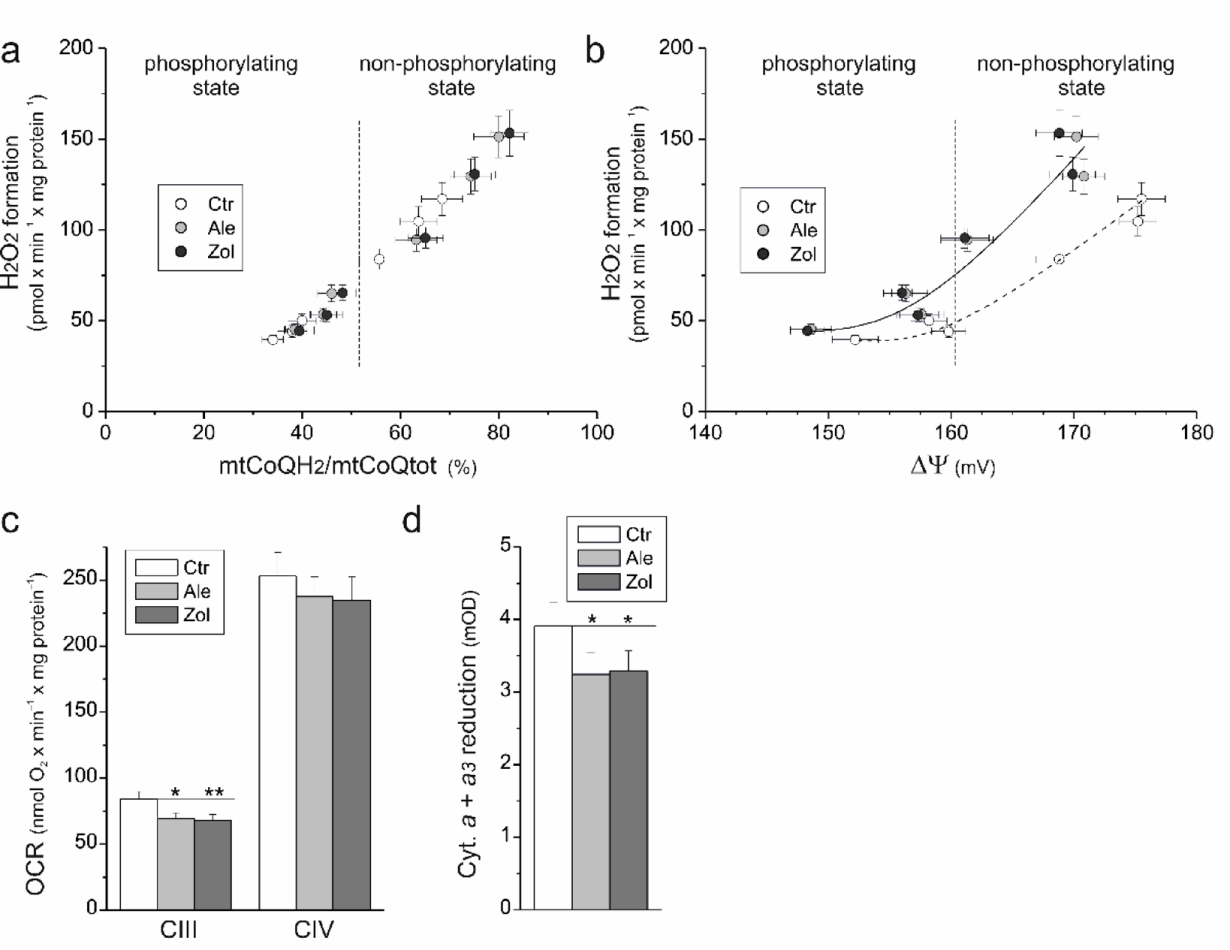
Relationships between (**a**) H_2_O_2_ production and the mtCoQ reduction level and (**b**) H_2_O_2_ production and ΔΨ of the mitochondria isolated from control (Ctr) and alendronate (Ale)- or zoledronate (Zol)-treated endothelial cells during succinate (with rotenone), malate, and a mixture of succinate and malate oxidation under non-phosphorylating and phosphorylating conditions. (**c**) Maximal CIII activity and CIV activity. (**d**) Reduction of cytochromes *a* + *a*_*3*_. Mean ± SD; *n* = 4–6; statistics: one-way ANOVA, *P* < 0.01 (**); *P* < 0.05 (*) relative to control mitochondria (horizontal lines; **c**, **d**).

Regarding the oxidation of the tested substrates under non-phosphorylating and phosphorylating conditions by the mitochondria of N-BP-treated and control cells, a single relationship was obtained between H_2_O_2_ production and the level of mtCoQ reduction (Fig. 4a), confirming the direct dependence of mtROS generation on the level of mtCoQ reduction^39^. The relationship between ΔΨ and the mtCoQ reduction level obtained for the mitochondria of N-BP-treated cells shifted relative to the mitochondria of control cells to values with higher H_2_O_2_ production and lower ΔΨ (Fig. 4b), indicating inhibition of the mtCoQH_2_-oxidizing pathway. To investigate further, we analyzed the activities of CIII and CIV. In the mitochondria of N-BP-treated cells, CIII activity was ~ 20% lower than that in the mitochondria of control cells, whereas CIV activity was unaffected (Fig. 4c). However, in the mitochondria of N-BP-treated cells, the level of reduction of cytochromes *a* + *a*_*3*,_ components of CIV, was ~ 17% lower than that in the mitochondria of control cells (Fig. 4d). The latter result indicates that in the mitochondria of endothelial cells treated with N-BPs, these mevalonate pathway inhibitors may decrease the level of heme *a*, another product of the pathway in addition to CoQ. However, this result requires further investigation.

Our results indicate that the overall increase in mtROS production is due to the increased level of mtCoQ reduction (mtCoQH_2_/mtCoQtot) resulting from a significant decrease in the size of the mtCoQ pool in the mitochondria of N-BP-treated endothelial cells. To understand why, in the mitochondria of N-BP-treated endothelial cells, oxidation of the CII substrate caused greater decreases in respiration and ΔΨ but smaller increases in mtROS production and mtCoQ reduction level compared with oxidation of the CI substrate, we further examined the N-BP-induced changes in the activity and levels of individual components of the OXPHOS system and its molecular organization.

### N-BPs change the molecular organization of the OXPHOS system in the mitochondria of endothelial cells, downregulating CIII and CIV in the CIII_2_ + CIV supercomplex and CII, CIII_2_, and CV

**Fig. 5:**
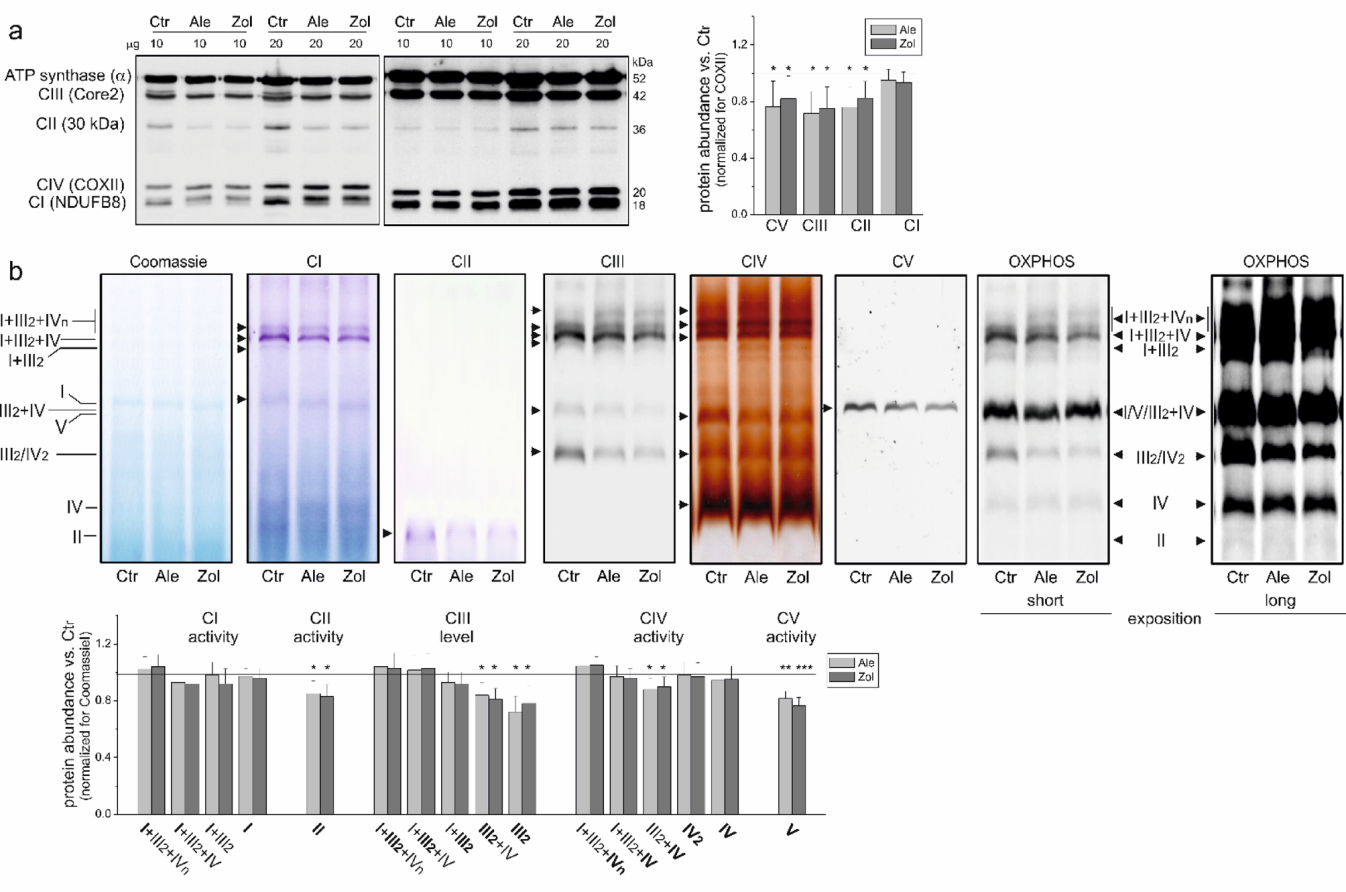
(**a**) OXPHOS complexes and (**b**) supercomplexes in the mitochondria isolated from control (Ctr) and alendronate (Ale)- or zoledronate (Zol)-treated endothelial cells. (**a**) Representative immunoblots and analysis of protein abundance. Mean ± SD; *n* = 6; statistics: one-way ANOVA, *P* < 0.05 (*) relative to control mitochondria. (**b**) Representative BN‒PAGE showing OXPHOS supercomplexes and complexes, and analysis of changes in in-gel activity or protein levels. Coomassie staining, CI and CII in-gel activity, CIII immunoblotting, CIV and CV in-gel activity, and total OXPHOS immunoblotting (short and long exposition) are shown in sequence. Mean ± SD; *n* = 4; statistics: KW ANOVA, *P* < 0.001 (***); *P* < 0.01 (**); *P* < 0.05 (*) relative to control mitochondria. The gels and original immunoblots are shown in Fig. S3 (Supplementary Information). CI, CII, CIII, CIV, respiratory chain complexes; CV, F_0_F_1_ ATP synthase.

Immunodetection of OXPHOS components revealed an ~ 20% reduction in the abundance of CII (subunit SDHB), CIII (subunit Core 2), and ATP synthase (subunit α) in the mitochondria of N-BP-treated endothelial cells (Fig. 5a). In contrast, the protein levels of CI (subunit NDUFB8) and CIV (subunit COXII) remained unchanged (Fig. 5a). Furthermore, BN-PAGE followed by in-gel activity assays revealed that CII and CV (ATP synthase) activities decreased after exposing endothelial cells to alendronate or zoledronate (Fig. 5b). In contrast, CI activities in all CI-associated supercomplexes remained unchanged (Fig. 5b).

In the mitochondria of N-BP-treated endothelial cells, while the protein levels of CI + CIII-associated supercomplexes (I + III_2_ + IV_(n)_ and I + III_2_) did not differ from those in the mitochondria of control cells, the levels of the III_2_ + IV supercomplex and III_2_ dimer decreased (Fig. 5b). These observations are consistent with the decreased level of CIII (subunit Core 2) observed via SDS‒PAGE (Fig. 5a) and with the decreased CIII activity, measured during the oxidation of duroquinol (Fig. 4c) in intact mitochondria of N-BP-treated endothelial cells. Furthermore, the in-gel activity of CIV in the III_2_ + IV supercomplex decreased in the mitochondria of N-BP-treated endothelial cells, whereas the activity of other CIV-associated supercomplexes/complexes and the protein level of CIV (COXII subunit) did not change (Fig. 5). However, in intact mitochondria of N-BP-treated endothelial cells, CIV activity remained unchanged (Fig. 4c), although the levels of the III_2_ + IV supercomplex (Fig. 5b) and cytochromes *a* + *a*_*3*_ (Fig. 4d) also decreased.

These results indicate that chronic exposure to N-BPs, which leads to a significant decrease in the level of mtCoQ in endothelial mitochondria, causes a rearrangement of the molecular organization of the respiratory chain supercomplexes (i.e., a decrease in the level of the III_2_ + IV supercomplex with an accompanying decrease in the protein level and activity of CII, CIII, and CV). These changes may lead to more effective use of deficient mtCoQ in the mitochondria of endothelial cells treated with N-BPs.

### In N-BP-treated endothelial cells, mitochondrial UCP2 expression is increased

**Fig. 6:**
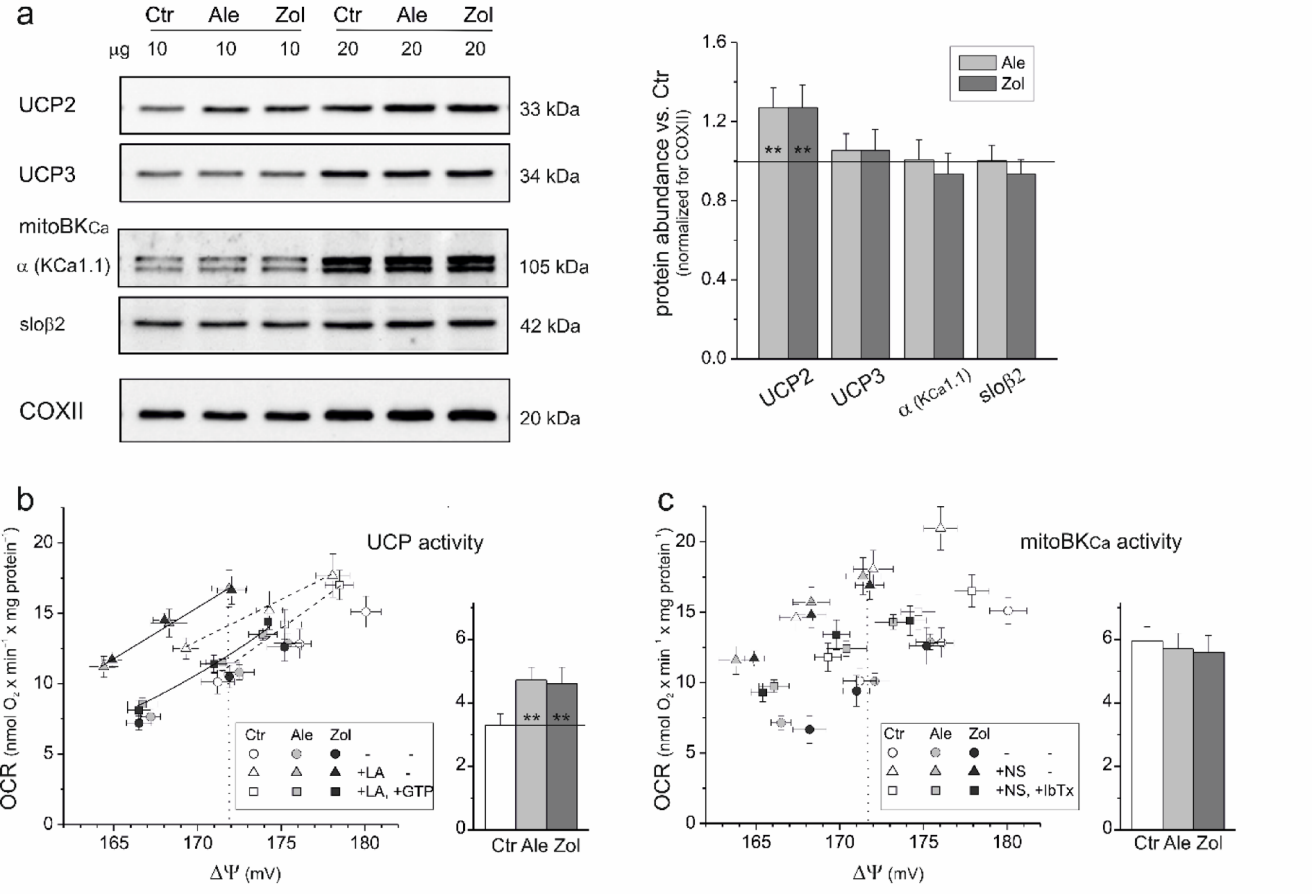
(**a**) Protein levels and (**b**) activities of energy-dissipating systems, UCP and mitoBK_Ca_, in the mitochondria of control (Ctr) and alendronate (Ale)- or zoledronate (Zol)-treated endothelial cells. (**a**) Analyses of protein abundance accompanied by representative immunoblots. Mean ± SD; *n* = 6; statistics: one-way ANOVA, *P* < 0.01 (**) relative to control mitochondria. Subunits of mitoBK_Ca_: α (K_Ca_1.1), pore α subunit K_Ca_1.1, and sloβ2, auxiliary sloβ2 subunit. The original immunoblots can be found in Figs. S4, S5 (Supplementary Information). (**b**) Linoleic acid (LA)-induced GTP-inhibited UCP-mediated H^+^ leakage (UCP activity). **c**, NS11021 (NS)-induced IbTx-inhibited mitoBK_Ca_ activity. (**b**, **c**) Relationships between the oxygen consumption rate (OCR) and ΔΨ for rotenone + malonate titration during the oxidation of a malate-succinate mixture under non-phosphorylating conditions. UCP and mitoBK_Ca_ activity were determined at the same ΔΨ (~ 172 mV). Mean ± SD; *n* = 4; statistics: KW ANOVA, *P* < 0.01 (**) relative to control mitochondria.

The question arises whether, owing to the increased mtROS production by the mitochondria of endothelial cells treated with alendronate or zoledronate (Fig. 3c), N-BPs affect the mitochondrial energy-dissipating systems, UCP and mitoBK_Ca_, which may act as antioxidant systems by increasing electron flow in the respiratory chain^33,40,41^. UCP2 expression was ~ 27% higher in the mitochondria of N-BP-treated endothelial cells than in the mitochondria of control cells, whereas UCP3 expression was unchanged (Fig. 6a). The protein levels of the mitoBK_Ca_ subunits, including the pore α subunit and regulatory sloβ2 subunit (found in endothelial mitochondria^42^ were also unaffected in the mitochondria of N-BP-treated cells (Fig. 6a).

Moreover, the flux‒force kinetics assay revealed increased fatty acid-induced GTP-inhibited UCP activity (~ 40%) and unchanged NS11021-induced IbTx-inhibited mitoBK_Ca_ activity (Fig. 6b and c) in the mitochondria of N-BP-treated cells compared with the mitochondria of control cells, confirming the immunodetection results (Fig. 6a). The increase in UCP activity can be linked to UCP2, as its protein levels were elevated. When the activity of mtCoQ-reducing dehydrogenases (CI and CII) was reduced (by titration with rotenone and malonate), ΔΨ depolarization was accompanied by increased sensitivity of UCP activity to GTP. These results support that lower mtCoQ reduction enhances UCP activity inhibition by purine nucleotides^40,43^.

These findings indicate that the mitochondria of N-BP-exposed endothelial cells exhibit higher levels of UCP2 expression and activity than the mitochondria of control cells, which may reduce mtROS production under conditions of mtCoQ deficiency.

## Discussion

We have previously shown that 5 µM alendronate and 1 µM zoledronate cause a similar decrease in the cellular level of CoQ in endothelial cells without affecting cell viability^19^. The effects of such concentrations of these N-BPs on the bioenergetic function of endothelial mitochondria were also similar in the present study.

A comparison of mitochondria isolated from human endothelial cells cultured for six days with or without 5 µM alendronate or 1 µM zoledronate revealed a significant 45–50% decrease in mtCoQ content (Fig. 1a) caused by these inhibitors of a crucial enzyme of the mevalonate pathway. Thus, inhibition of the mevalonate pathway by N-BPs, which disrupts the synthesis of the isoprenoid chain necessary for CoQ biosynthesis^12,13^, leads to reduced mtCoQ levels. Interestingly, the observed decrease in CoQ10B levels (Fig. 1b) also indicates potential changes in the CoQ biosynthetic pathway downstream of the mevalonate pathway^44^. However, further analyses are necessary to fully elucidate the regulatory mechanisms of the CoQ biosynthetic pathway under N-BP exposure, including other CoQ proteins essential for CoQ biosynthesis^44^ at the transcriptional and posttranscriptional levels.

This study is the first to demonstrate an N-BP-induced decrease in mtCoQ levels in mitochondria. There is also little research on the effects of N-BPs on cellular CoQ levels. Plasma CoQ levels are decreased in postmenopausal women treated with N-BPs^18^. Our previous studies revealed an ~ 30% decrease in total CoQ levels (i.e., CoQ from all membranes) in endothelial cells cultured with alendronate or zoledronate^19^. The current study revealed that in the mitochondria of N-BP-treated endothelial cells, mtCoQ deficiency was accompanied by the loss of the pool of mtCoQH_2_, which has an antioxidant function, and accounts for ~ 12% of the total mtCoQ pool in the mitochondria of untreated endothelial cells (Fig. 1a). The N-BP-induced loss of the mtCoQH_2_ antioxidant pool was accompanied by increased levels of the antioxidant enzyme SOD2 and increased UCP2 levels and activity (Figs. 1b and 6a and b). Our previous studies demonstrated that UCP2 might play a physiological role in reducing mtROS formation during conditions of heightened oxidative stress in endothelial cells, such as exposure to high levels of glucose^45^ and palmitic acid^46^, and to statins, which, like N-BPs, block the mevalonate pathway^15^.

The N-BP-induced 45–50% decrease in mtCoQ content (Fig. 1a) indicates a significantly lower availability of mtCoQ in the mitochondrial membrane, which may strongly affect the efficiency of the respiratory chain and ATP synthesis and impact mtROS formation, potentially affecting overall cellular metabolism. In endothelial mitochondria, N-BPs led to an overall reduction in the maximal oxidation of respiratory substrates (malate, succinate, and glutamate), whereas the oxidation of the least oxidized palmitoylcarnitine increased (Fig. 2). These findings suggest that N-BPs reduce the capacity of endothelial mitochondria to metabolize intermediates effectively in the TCA cycle. Furthermore, endothelial cells may adapt to reduced energy availability from glucose or amino acid oxidation by relying more on fat-derived energy sources. Our results show that N-BPs modulate ACADS, an enzyme involved in fatty acid oxidation, by increasing its levels (Fig. 2b). Previous studies have shown that endothelial cells exposed to N-BPs are less able to efficiently utilize glucose and amino acid substrates but demonstrate increased fatty acid oxidation^19^. In the mitochondria of N-BP-treated cells, decreased substrate flux into the electron transport chain leads to decreased ATP production, as indicated by a decreased ADP/O ratio, ADP phosphorylation rate (Table 1), and ATP synthase activity level (Fig. 5b). Fatty acids provide more ATP per molecule than carbohydrates do; thus, cells may attempt to optimize energy production under N-BP exposure by switching to a more energy-rich fuel source. Interestingly, the significant reduction in the mtCoQ pool (Fig. 1a) in the mitochondria of N-BP-treated cells was accompanied by a decrease in the activity and protein level of CII, not CI (Fig. 5). Oxidation of the CI substrate provides more ATP than does oxidation of the CII substrate (higher rate of ADP phosphorylation; Table 1). Thus, mitochondria may attempt to maintain energy production under N-BP exposure by limiting only the inflow of electrons from CII, not CI, to the diminished mtCoQ pool.

The decrease in the mtCoQ pool in mitochondria from N-BP-exposed endothelial cells resulted in a decline not only in mtCoQ-reducing CII (Fig. 5) but also in the activity and level of CIII that oxidizes QH_2_ (Figs. 4c and 5). However, the decrease in the maximal activity of CIII in intact mitochondria was accompanied by rearrangements at the organizational level of its supercomplexes. The CIII protein level and the CI activity of the CI + CIII-related supercomplexes (I + III_2_ + IV(n) and I + III_2_) did not change, whereas the levels of the III_2_ + IV supercomplex and the III_2_ dimer decreased (Fig. 5b). These changes may serve to more effectively use the deficient mtCoQ and maintain electron flow through the CI + CIII + CIV pathway in the mitochondria of N-BP-treated endothelial cells. Previous studies have shown that statin- or hypoxia-induced mtCoQ deficiency also causes a specific rearrangement of the molecular organization of OXPHOS system components in endothelial mitochondria^15,33^.

Our results indicate that in the mitochondria of cells treated with N-BPs, these mevalonate pathway inhibitors may decrease the level of *a*-heme (cytochromes *a* + *a*_*3*_) (Fig. 4d), the prosthetic group of CIV. However, further studies are needed to investigate whether inhibition of the mevalonate pathway by N-BPs could impair the synthesis of the isoprenoid chain required for the biosynthesis of heme *a*, a key component of mitochondrial COX (CIV), potentially leading to reduced cytochrome *a* levels and impaired mitochondrial respiratory function. However, in our study, despite reduced *a*-heme levels in the CIV (Fig. 4d), no effect on the maximal activity of this complex was observed in the intact mitochondria of N-BP-treated endothelial cells compared with that of the mitochondria of control cells (Fig. 4c). Only the in-gel activity of CIV in the III_2_ + IV supercomplex decreased in the mitochondria of N-BP-treated cells (Fig. 5b). The maximal activity of CIV (~ 250 nmol O_2_ × min^−1^ × mg protein^−1^; Fig. 4c) is not a factor limiting the flow of electrons in the respiratory chain in endothelial mitochondria, because with the activity of both CI and CII dehydrogenases, the maximal activity of the chain is much lower (~ 70 nmol O_2_ × min^−1^ × mg protein^−1^; Fig. 3a).

Our findings demonstrate that prolonged exposure of endothelial cells to alendronate or zoledronate modulates the activity and composition of the mitochondrial respiratory chain and slows OXPHOS. Moreover, the significant N-BP-induced ~ 45–50% decrease in mtCoQ content (Fig. 1a) resulted in increased mtCoQ reduction (mtCoQH_2_/mtCoQtot) under both phosphorylating and non-phosphorylating conditions (Fig. 3d), resulting in increased mtROS formation (Fig. 3c). The decreases in the mtCoQ pool and the activity of the mtCoQH_2_-oxidizing pathway (CIII + CIV) related to decreased CIII activity may account for the increased reduction in mtCoQ, resulting in a general increase in mtROS production in the mitochondria of N-BP-treated endothelial cells. The decrease in CII activity, along with the stable CI activity (Fig. 5), accounts for the more significant decreases in respiration and ΔΨ (Figs. 3a, b) and the less pronounced increases in mtROS production and mtCoQ reduction (Figs. 3c, d) observed during CII substrate oxidation than during CI substrate oxidation. Decreasing mtCoQ-reducing dehydrogenase (CII) activity decreases the level of mtCoQ reduction and therefore mtROS formation. Decreasing mtCoQH_2_-oxidizing CIII activity (pronounced during phosphorylating respiration) increases the level of mtCoQ reduction and mtROS production. Moreover, the inhibition of the mtCoQH_2_-oxidizing pathway is clearly indicated by the shift in the relationship between ΔΨ and the mtCoQ reduction level, obtained for the mitochondria of N-BP-treated cells relative to the mitochondria of control cells, to values with higher ROS production and lower ΔΨ (Fig. 4b). Thus, our results indicate that exposing endothelial cells to N-BPs alters mtCoQ redox homeostasis, a key factor modulating mtROS production^1,39^. The increased mtCoQ reduction level induced by N-BPs and, consequently, the increased mtROS production (Figs. 3c, d) were accompanied by elevated levels of the antioxidant enzymes SOD2 and UCP2 (Figs. 1 and 6). For the first time, our study correlates changes in mtCoQ content, mtCoQ reduction level, and mtROS production in the mitochondria of endothelial cells exposed to N-BPs.

The observed disruption of mtCoQ homeostasis by N-BPs has important implications for endothelial pathophysiology, particularly in the context of cardiovascular disease, in which mitochondrial dysfunction and oxidative stress contribute to the onset and progression of the disease^20–23,47,48^. Decreased mtCoQH_2_ levels directly impair the mitochondrial antioxidant defense system, leading to increased ROS production, which may activate redox-sensitive signaling pathways involved in endothelial activation, inflammation, and apoptosis. These mechanisms are closely related to the initial stages of atherosclerosis and microcirculation dysfunction^20,49,50^. In addition, reduced expression of key respiratory complexes (CII, CIII, and CV) and remodeling of supercomplexes may contribute to bioenergetic failure and induce metabolic reprogramming in endothelial cells, favoring fatty acid oxidation over glucose metabolism, a phenotype observed in endothelial cells under conditions of chronic stress or aging^15,19,45,51–54^. Targeting these mitochondrial vulnerabilities, for example through CoQ supplementation, represents a promising therapeutic avenue. Further studies should aim to validate these mechanisms in in vivo models and clinical settings.

Although N-BP drugs are commonly used to treat osteoporosis and other bone diseases, evidence suggests that long-term N-BP or high-dose N-BP therapy may have unintended effects on extraskeletal tissues, including the endothelium^24–27^. Although the primary aim of our study was to analyze the bioenergetic effects of N-BPs on endothelial mitochondria, the disturbances in mitochondrial function and mtCoQ redox homeostasis we observed may have broader implications for cardiovascular health. We propose that N-BP-induced mitochondrial dysfunction, particularly through mtCoQ deficiency/depletion and the resulting increase in mtROS, may contribute to the development of endothelial dysfunction and increase the risk of cardiovascular disease especially in individuals receiving long-term N-BP therapy. However, while this study provides important mechanistic insights at the mitochondrial level, further research is needed to determine the clinical relevance of these findings. Further studies are needed to investigate the systemic cardiovascular effects of N-BPs in animal models or clinical populations to better elucidate the full extent of these effects and their clinical implications. Furthermore, future studies should evaluate whether supplementation with exogenous CoQ, particularly in its bioavailable form (e.g., mitochondria-targeted CoQ10), can counteract N-BP-induced mitochondrial dysfunction and protect endothelial function, confirming the role of mtCoQ deficiency in the observed mitochondrial dysfunction and redox imbalance.

## Conclusions

Our study revealed that chronic exposure to N-BPs, which are commonly used to treat osteoporosis, significantly affects mitochondrial bioenergetic functions in human endothelial cells, primarily by dramatically reducing the mtCoQ content. In N-BP-treated endothelial cells, a significant decrease in mtCoQ content was accompanied by an overall decrease in mitochondrial respiratory activity and OXPHOS efficiency, as well as a more reduced redox state of mtCoQ, leading to increased mtROS production. Moreover, changes at the molecular organization level of the OXPHOS system and a greater share of fatty acid oxidation were observed. Understanding the N-BP-induced adaptation of mitochondrial bioenergetics associated with mtCoQ deficiency may shed light on the molecular mechanisms underlying potential N-BP-induced endothelial dysfunction. This study also provides information to support strategies to mitigate potential side effects, possibly through CoQ10 supplementation. Because mitochondria are potential sites of pharmacological intervention aimed at broadly understood cell protection against oxidative stress, our research may prove helpful in verifying or supplementing (e.g., through CoQ10 supplementation) existing N-BP therapies. This study highlights the importance of conducting further research on the effects of N-BPs on endothelial mitochondria and the overall energy metabolism of endothelial cells.

## Electronic supplementary material

Below is the link to the electronic supplementary material.

Supplementary Material 1

## Data Availability

Data is provided within the manuscript or supplementary information files.

## Abbreviations

ACADS: Acyl-coenzyme A dehydrogenase
CoQ: Coenzyme Q
CoQ10: Coenzyme Q10
CoQ10B: Coenzyme Q-binding protein CoQ10 homolog B
CS: Citrate synthase
COX: Cytochrome *c* oxidase, complex IV
CI-CIV: Complexes of respiratory chain
FPPS: Farnesyl diphosphate synthase
GDH: Glutamate dehydrogenase
mitoBK_Ca_: Mitochondrial large-conductance Ca^2+^-activated potassium channel
mtCoQ: Mitochondrial CoQ
mtCoQH_2_/mtCoQtot: MtCoQ reduction level (reduced mtCoQ/total mtCoQ)
N-BP(s): Nitrogen-containing bisphosphonates
OXPHOS: Oxidative phosphorylation
ROS: Reactive oxygen species
mtROS: Mitochondrial ROS
SOD2: Superoxide dismutase 2
UCP(2, 3): Uncoupling protein (2,3)
ΔΨ: Mitochondrial transmembrane electrical potential
