# Supplementary material for: Adaptation of mitochondrial bioenergetics to coenzyme Q deficiency in human endothelial cells after chronic exposure to bisphosphonates
Source: Sci Rep. 2025 May 22;15:17734. doi: 10.1038/s41598-025-02710-8 (PMC12098996; doi:10.1038/s41598-025-02710-8)
Supplement: Supplementary file 1 — Supplementary Material 1 [file 41598_2025_2710_MOESM1_ESM.pdf]

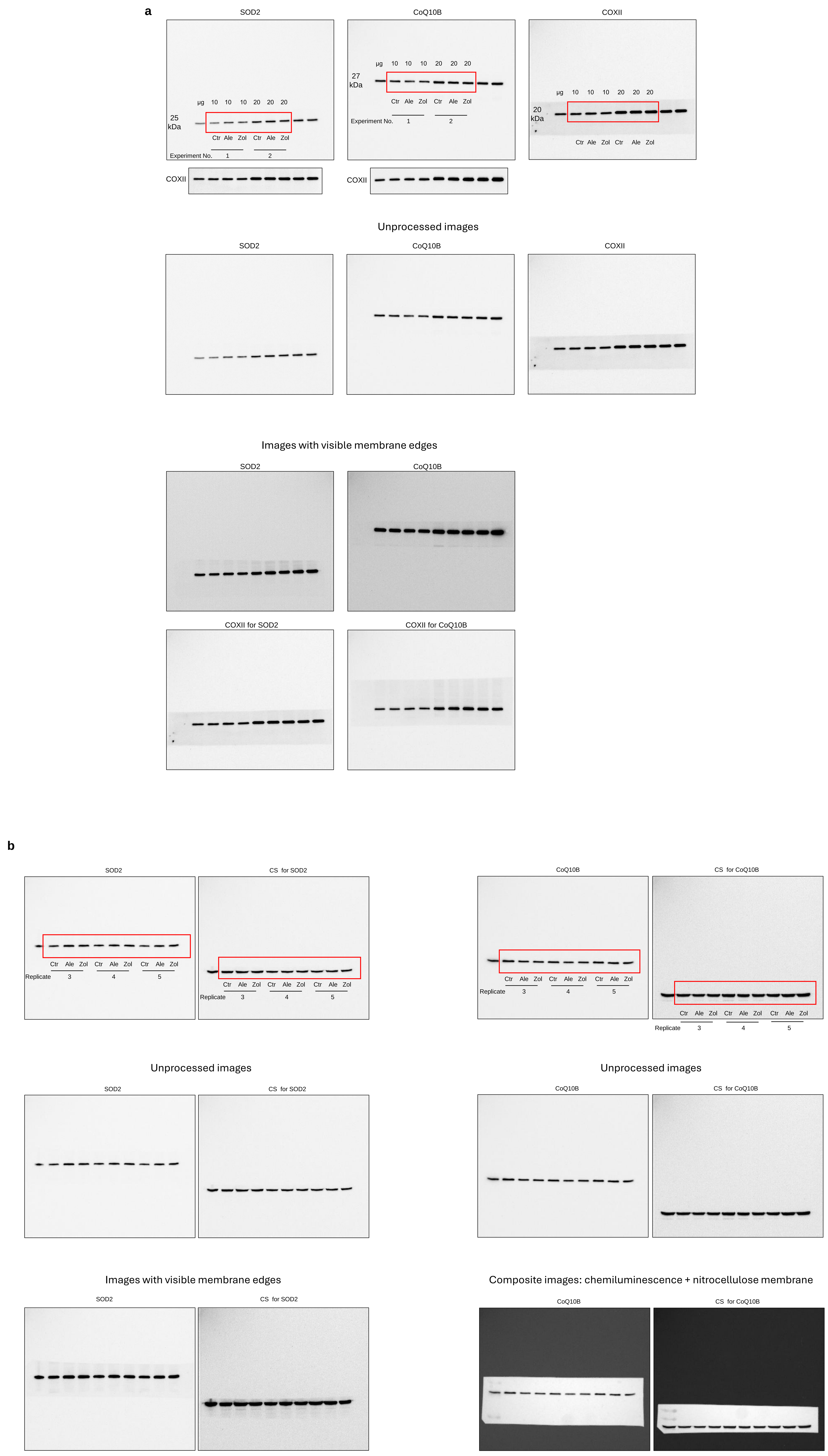


**Supplementary Fig. S1.** Uncropped images of blots shown in Fig. 1b (**a**) and images used for densitometric analysis (**b**). Ctr, control mitochondria; Ale, mitochondria of alendronate-treated cells; Zol, mitochondria of zoledronate-treated cells.

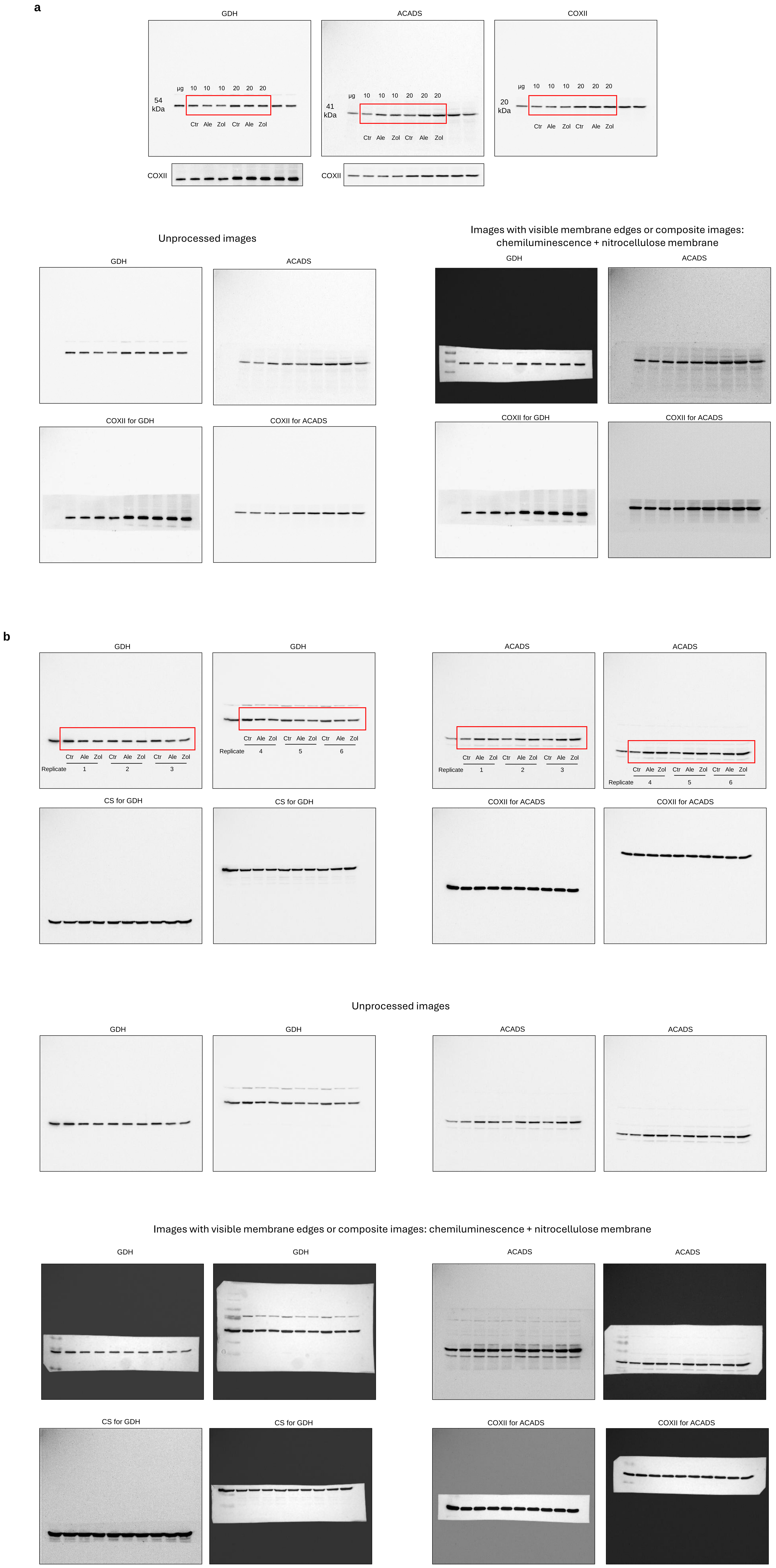


**Supplementary Fig. S2.** Original images of western blots used to prepare Fig. 2b (**a**) and blots used for densitometric analysis (**b**). Ctr, control mitochondria; Ale, mitochondria of alendronate-treated cells; Zol, mitochondria of zoledronate-treated cells.

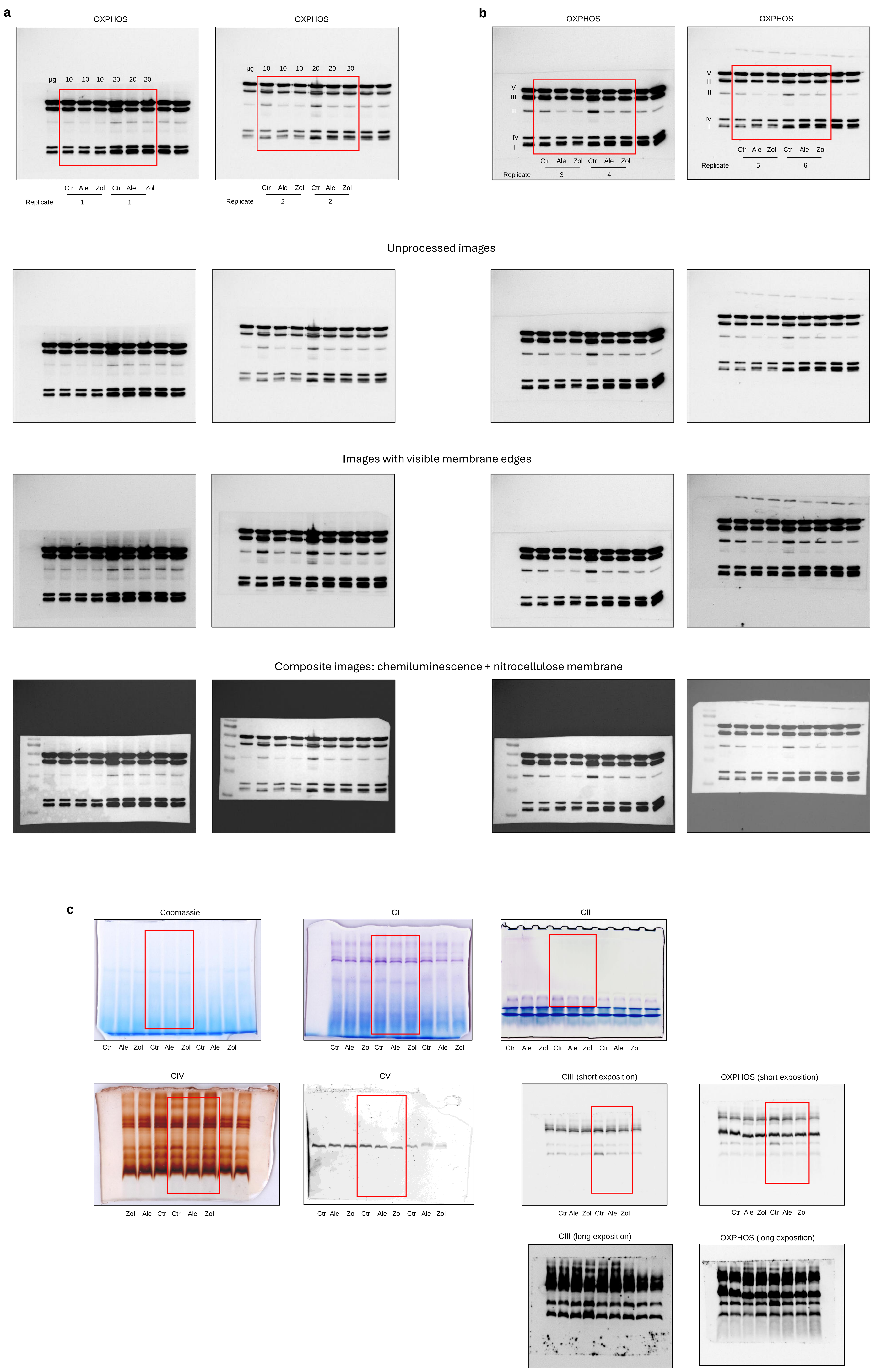


**Supplementary Fig. S3.** Images used to prepare Fig. 5. **a,** Unprocessed images of blots shown in Fig. 5a. **b,** Images used to perform the calculations shown in Fig. 5a. **c,** Uncropped images of gels and blots presented in Fig. 5b. Ctr, control mitochondria; Ale, mitochondria of alendronate-treated cells; Zol, mitochondria of zoledronate-treated cells.

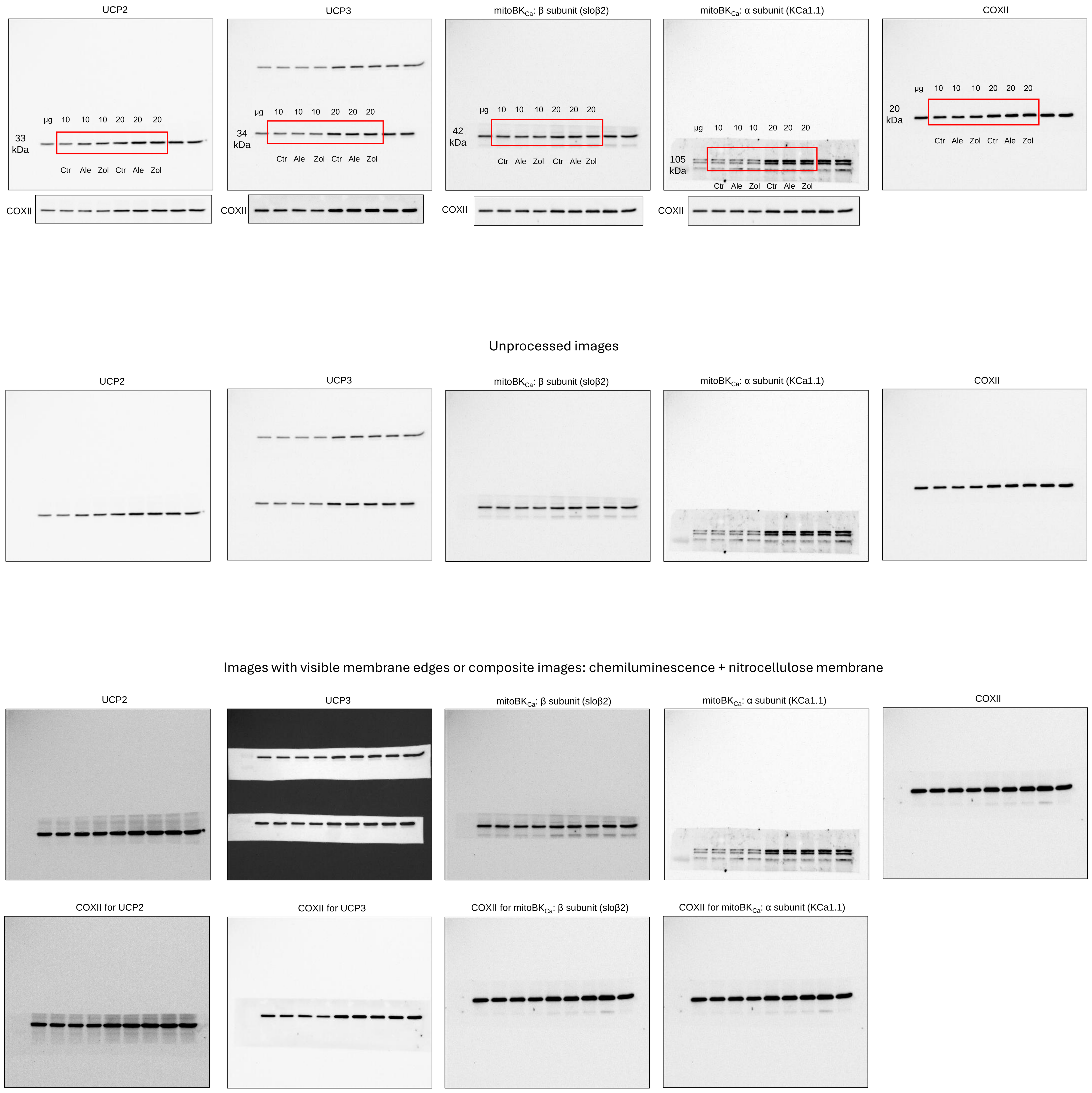


**Supplementary Fig. S4**. Uncropped images of blots shown in Fig. 6a. Ctr, control mitochondria; Ale, mitochondria of alendronate-treated cells; Zol, mitochondria of zoledronate-treated cells.

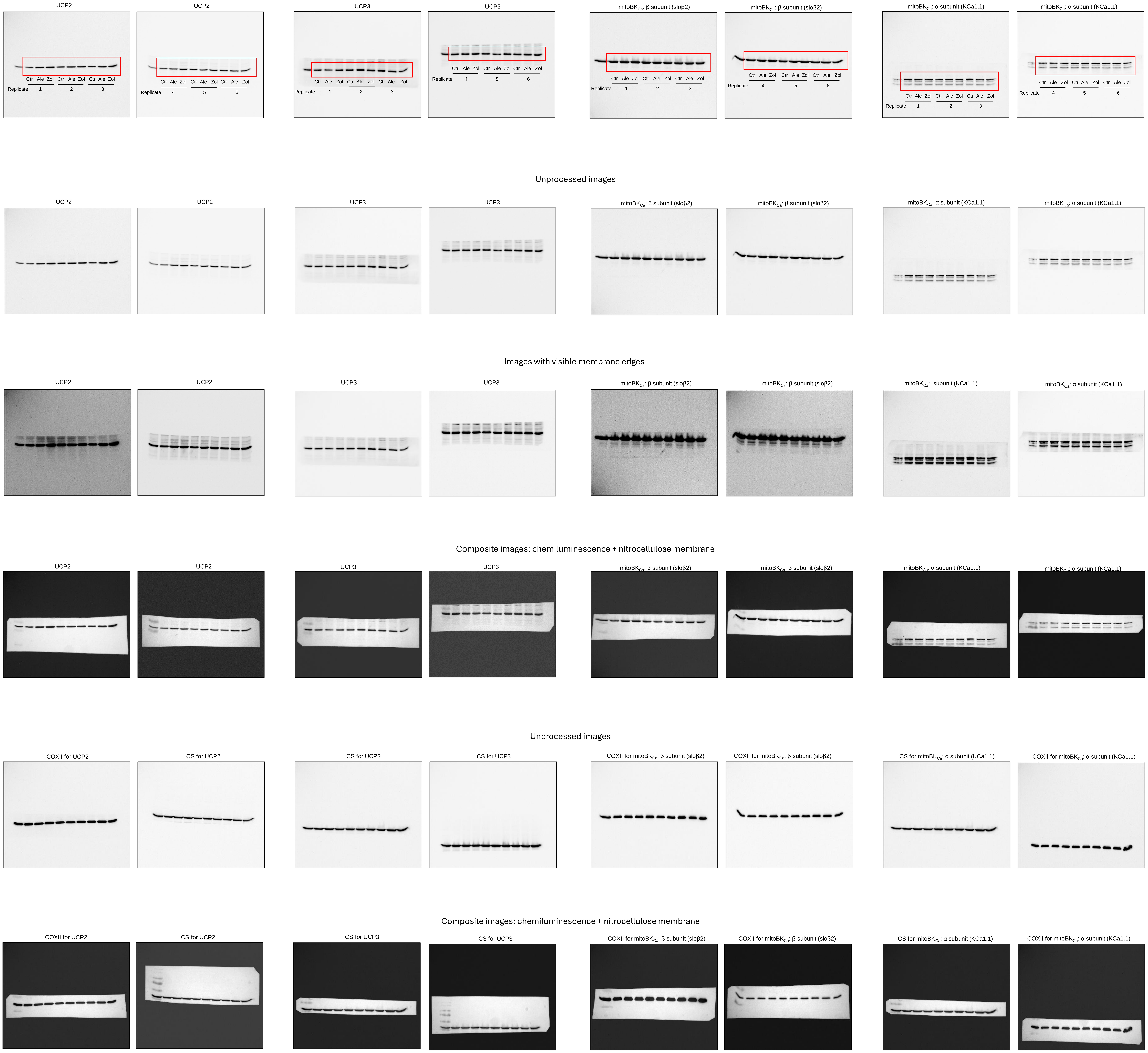


**Supplementary Fig. S5**. Images used for densitometric analysis shown in Fig. 6a. Ctr, control mitochondria; Ale, mitochondria of alendronate-treated cells; Zol, mitochondria of zoledronate-treated cells.
